# Efficacy and Safety of Metronidazole Monotherapy versus Vancomycin Monotherapy or Combination Therapy in Patients with *Clostridium difficile* Infection: A Systematic Review and Meta-Analysis

**DOI:** 10.1371/journal.pone.0137252

**Published:** 2015-10-07

**Authors:** Rui Li, Laichun Lu, Yu Lin, Mingxia Wang, Xin Liu

**Affiliations:** 1 College of Pharmacy, Chongqing Medical University, Chongqing, China; 2 Department of Pharmacy, Third Affiliated Hospital, Third Military Medical University, Chongqing, China; Cleveland Clinic, UNITED STATES

## Abstract

**Background:**

*Clostridium difficile* infection (CDI) has become a global epidemiological problem for both hospitalized patients and outpatients. The most commonly used drugs to treat CDI are metronidazole and vancomycin. The aim of this study was to compare the efficacy and safety of metronidazole monotherapy with vancomycin monotherapy and combination therapy in CDI patients.

**Methods:**

A comprehensive search without publication status or other restrictions was conducted. Studies comparing metronidazole monotherapy with vancomycin monotherapy or combination therapy in patients with CDI were considered eligible. Meta-analysis was performed using the Mantel-Haenszel fixed-effects model, and odds ratios (ORs) with 95% confidence intervals (95% CIs) were calculated and reported.

**Results:**

Of the 1910 records identified, seventeen studies from thirteen articles (n = 2501 patients) were included. No statistically significant difference in the rate of clinical cure was found between metronidazole and vancomycin for mild CDI (OR = 0.67, 95% CI (0.45, 1.00), p = 0.05) or between either monotherapy and combination therapy for CDI (OR = 1.07, 95% CI (0.58, 1.96), p = 0.83); however, the rate of clinical cure was lower for metronidazole than for vancomycin for severe CDI (OR = 0.46, 95% CI (0.26, 0.80), p = 0.006). No statistically significant difference in the rate of CDI recurrence was found between metronidazole and vancomycin for mild CDI (OR = 0.99, 95% CI (0.40, 2.45), p = 0.98) or severe CDI (OR = 0.98, 95% CI (0.63, 1.53), p = 0.94) or between either monotherapy and combination therapy for CDI (OR = 0.91, 95% CI (0.66, 1.26), p = 0.56). In addition, there was no significant difference in the rate of adverse events (AEs) between metronidazole and vancomycin (OR = 1.18, 95% CI (0.80, 1.74), p *=* 0.41). In contrast, the rate of AEs was significantly lower for either monotherapy than for combination therapy (OR = 0.30, 95% CI (0.17, 0.51), p<0.0001).

**Conclusions:**

Metronidazole and vancomycin are equally effective for the treatment of mild CDI, but vancomycin is superior for the treatment of severe CDI. Combination therapy is not superior to monotherapy because it appears to be associated with an increase in the rate of AEs.

## Introduction

*Clostridium difficile* is an anaerobic, gram-positive, spore-forming bacillus[1]. The incidence and severity of *C*. *difficile* infection (CDI) appear to be on the rise[2], and CDI has become an epidemiological problem for both hospitalized patients and outpatients worldwide[2].

Oral metronidazole is recommended for patients undergoing initial therapy and for those with mild infections, whereas oral vancomycin is recommended for seriously ill patients. Furthermore, the combination of oral vancomycin and intravenous metronidazole is suggested for the treatment of severe or complicated infections[3,4]. As risk of the development of vancomycin-resistant enterococci (VRE) associated with both metronidazole and vancomycin treatment has emerged[5], studies examining fidaxomicin[6,7], rifaximin[8,9], rifampin[10], nitazoxanide[11,12], tolevamer[13], and teicoplanin[14] therapies have been performed. Furthermore, in patients with recurrent CDI, infusion of donor feces has resulted in improved treatment outcomes. In particular, patients with multiple relapses of CDI have benefited from this approach[15]. Unfortunately, prospective clinical trials of most of the above-mentioned antibiotics evaluating their applicability for general use have not yet been performed[4], and several questions regarding fecal transplantation remain unanswered; for example, the optimal protocol for donor feces infusion is unknown[15]. Therefore, the treatment of CDI has relied primarily on metronidazole and vancomycin[16].

Although the Healthcare Epidemiology of America (SHEA)/Infectious Diseases Society of America (IDSA) guidelines recommend the use of oral vancomycin for severe CDI, this recommendation is based on the results of few clinical trials[17,18]. In addition, despite the frequent use of combination therapy in clinical settings, it is unclear whether combination therapy is more effective than monotherapy because, as emphasized by the European Society of Clinical Microbiology and Infectious Diseases (ESCMID)[4], few randomized controlled trials (RCTs) have been performed to compare them. Moreover, the many associated adverse events (AEs)[16] necessitate safety analyses of these two drugs. To address this knowledge gap, we performed a systematic review and meta-analysis to compare the efficacy and safety of metronidazole monotherapy with vancomycin monotherapy and combination therapy for the treatment of patients with CDI.

## Methods

Our study protocol and analysis were planned in accordance with the Preferred Reporting Items for Systematic Reviews and Meta-Analyses (PRISMA) guidelines[19], and no ethical approval was needed.

### Search strategy

A systematic literature search of the following electronic databases was conducted to identify relevant literature published in English or Chinese before November 2014: PubMed, Embase, Web of Science, and Cochrane Library. Three Chinese language databases—China National Knowledge Infrastructure (CNKI; available at www.cnki.net), Chinese Scientific Journals Database (VIP; available at www.cqvip.com), and WANFANG DATA (available at www.wanfangdata.com.cn)—were also searched. The search strategy included the following medical subject headings and key words: “pseudomembranous enterocolitis,” “antibiotic-associated diarrhea,” “antibiotic-associated colitis,” “*Clostridium difficile*,” “metronidazole,” and “vancomycin.” These search terms were combined using Boolean logic as follows: both the medical subject headings and the terms related to the patient population of interest (*Clostridium difficile*, pseudomembranous colitis, antibiotic-associated diarrhea, and antibiotic-associated colitis) were combined with those describing interventions (metronidazole OR vancomycin). The complete search strategy used to search each database is described in S1 Table. Furthermore, the references in the initially identified articles, including relevant reviews, were manually searched and reviewed. Academic dissertations and abstracts presented at scientific conferences were also searched using ProQuest Dissertation & Theses Database and The Conference and Academic Dissertation Database of CNKI, respectively, to ensure that no relevant study was missed (up to May 2015).

### Study selection

Two reviewers (RL and LCL) assessed all potentially relevant studies and reached a consensus on all items. They then independently searched the literature and examined relevant studies to obtain data on the rates of clinical cure, CDI recurrence and AEs. In the case of disagreement between the two reviewers, the senior coauthor (XL) was consulted, and the disagreement was resolved by consensus. Studies were included if they met the following criteria: (1) contained original data, (2) contained data regarding the use of metronidazole or vancomycin for the treatment of CDI, (3) contained data regarding clinical therapeutic outcomes or AEs, and (4) reported on RCTs or case-control studies. Studies were excluded if they (1) were unrelated to CDI, (2) were duplicate reports, (3) were not written in English or Chinese, or (4) were not case-control studies.

### Data extraction

Two reviewers (RL and LCL) independently abstracted data from each eligible study. Each investigator was blinded to the other investigator’s extracted data. A standardized form was used to record the following information: (1) first author; (2) year of publication; (3) disease severity (although there were four classifications according to disease severity, including mild CDI, severe CDI, complicated CDI, and recurrent CDI[3,4], none of the included studies evaluated complicated CDI, and fecal microbiota transplantation has been performed for recurrent CDI. Additionally, one included study referred to “mild-moderate” because the treatment of mild CDI and moderate CDI is identical[20], and we categorized this classification of CDI into the mild CDI group; thus, this systematic review and meta-analysis was primarily focused on the treatment of mild or severe CDI); (4) mean age; (5) male (%); (6) follow-up duration; (7) number of enrolled patients; (8) drug regimen; (9) rate of clinical cure; (10) rate of CDI recurrence; (11) AEs related to the study medications; (12) case definitions; (13) quality of the evidence; and (14) overall risk of bias. The extracted data (and specifically, the rates of clinical cure and CDI recurrence) were analyzed based on the intention-to-treat population.

### Quality appraisal

The included studies were independently appraised for methodological quality by two authors (RL and LCL), without blinding to the source journal or authorship. Discrepancies were resolved by discussion or consultation with the third reviewer (XL) if required. The quality of each included study was evaluated according to the modified Jadad score[21]. The overall risk of bias of each included study was also evaluated[22]. Potential publication bias was assessed by visual inspection of asymmetry in Begg’s funnel plots, and Egger’s test was then used to provide statistical evidence of funnel plot symmetry (p<0.05 indicating bias and p>0.05 not indicating bias)[23,24]. We also performed sensitivity analyses on subgroups from the studies according to explicit or inexplicit classification of severity and follow-up duration to explore underlying sources of heterogeneity. All of these analyses, except for publication bias analysis (which was performed using STATA software: version 12.0, StataCorp, College Station, TX, USA), were performed using Review Manager software (RevMan, version 5.1, Oxford, UK; The Cochrane Collaboration, 2008).

### Outcomes analyzed

Regarding the outcome measures used to assess efficacy, in this meta-analysis, the rate of clinical cure was used as the primary outcome measure, and the rate of CDI recurrence was used as the secondary outcome measure. We present the following two comparisons based on the rates of clinical cure and CDI recurrence: a comparison between metronidazole and vancomycin monotherapy for the treatment of patients with mild or severe CDI and a comparison of vancomycin or metronidazole monotherapy with combined vancomycin and metronidazole therapy or either drug combined with another antibiotic for the treatment of patients with CDI. For the latter comparison, we performed subgroup analysis according to the therapeutic intervention. AEs were used as the primary safety outcome measure in this meta-analysis. Here, we also present the following two comparisons based on AEs: metronidazole vs. vancomycin and monotherapy vs. combination therapy. We performed subgroup analysis according to AEs for both comparisons.

### Data analysis and statistical methods

Statistical analysis was accomplished using Review Manager version 5.1 software. Both I^2^ and Q statistics were considered. In our analyses, we used the I^2^ statistic to quantitatively describe heterogeneity across studies. An I^2^ value of greater than 50% indicated high heterogeneity; a value ranging from 25% to 50% indicated moderate heterogeneity; and a value of less than 25% signified low heterogeneity[22]. Pooled odds ratios (ORs; calculated by adding 0.5 to each cell of the 2×2 table for the trial when one arm of the study contained no events[25]) and 95% confidence intervals (CIs; if 95% CIs did not include the null value, the results were considered to be significantly different) were also used in meta-analysis. The Mantel-Haenszel fixed-effects model was used for analysis of the total groups because the I^2^ values in our subgroup meta-analysis ranged from 0% to 6%.

## Results

### Search results

**Fig 1:**
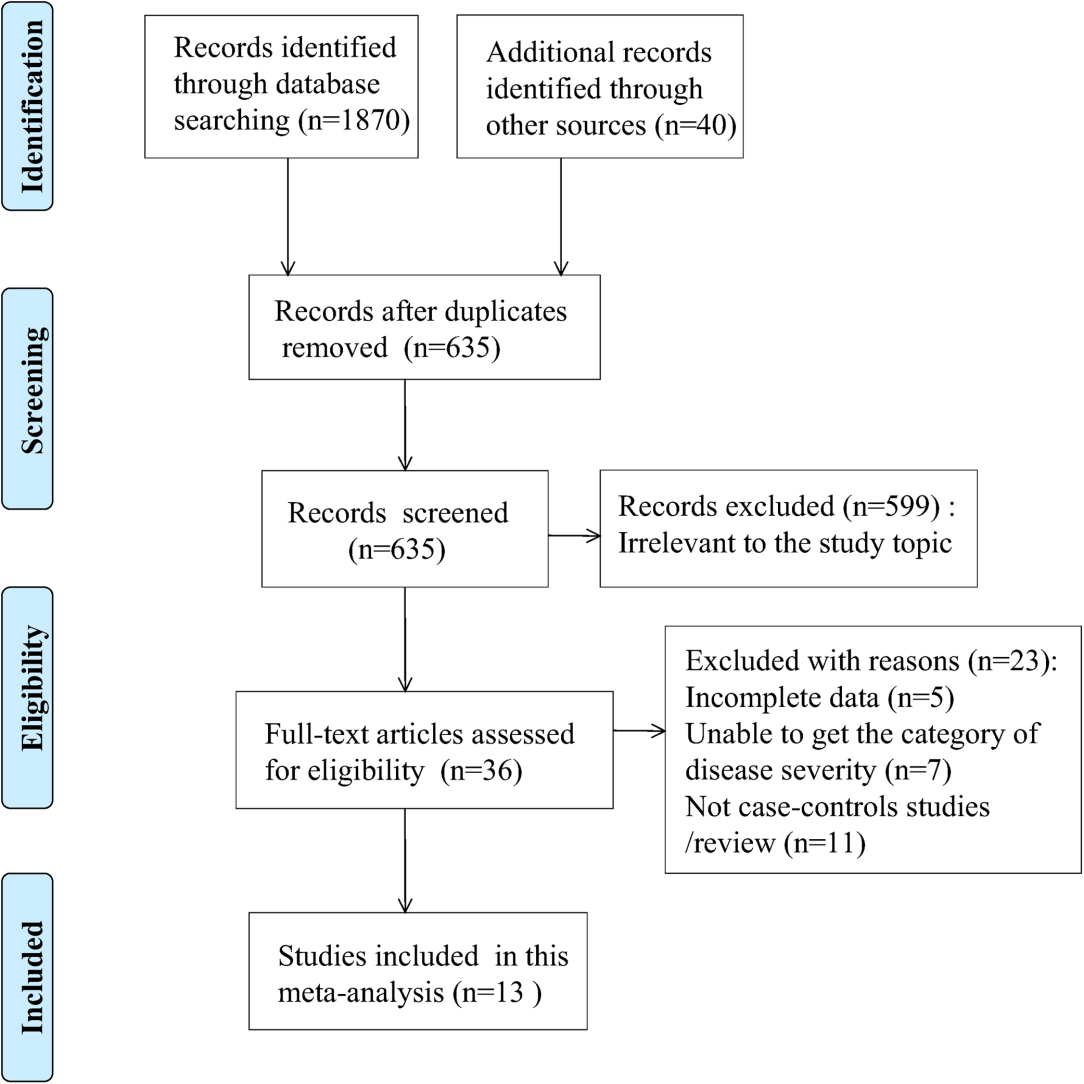
Flow chart depicting the selection process for the studies included in the meta-analysis.

A total of 1910 records were identified (Fig 1). After excluding duplicates and screening the titles of the studies, 635 articles were reviewed. After screening the abstracts of these potentially relevant articles, 36 were selected for full-text review based on relevance to the study topic (Fig 1). Thirteen articles containing seventeen studies (one article included three separate studies and two articles each included two separate studies) and 2501 patients were included[10,13,20,26–35]. In addition, we attempted to contact the corresponding authors of five potentially relevant studies via e-mail. The authors of four studies did not respond to our e-mail communication[36–39], and the corresponding author of the fifth study communicated that we would require approval by their institutional review board for additional individual patient data[40]. Therefore, all five studies had to be excluded from our final set of included studies. Fig 1 shows the selection process for the studies included in the meta-analysis.

### Study quality assessment and risk of bias assessment

Using the modified Jadad score (S2 Table), the results of the quality assessment of each included study indicated that five studies were of high quality[10,13,26,27,33] and that the remaining eight studies were of moderate quality[20,28–32,34,35] (Table 1, S2 Table). The results of the assessment of the overall risk of bias for each included study indicated that three reports exhibited a low risk of bias[10,13,26] and that the remaining ten reports exhibited an unclear risk of bias[20,27–35] (Table 1, S1 Fig). The main characteristics of the included studies are shown in Table 1.

**Table 1:** Main characteristics of the studies included in the meta-analysis.

Study	Disease Severity	MeanAge	Male(%)	Follow-up(weeks)	Enrolled Patients		Drug Regimen		Case Definitions	AssessmentIndex	EvidenceQuality	Risk of Bias
					T	C	T	C				
**Met vs. Van**												
Stuart,2014[13]	Mild/Severe	65.0	47.0	4	278	258	Met	Van	Method 1	(1)(3)	High	Low
Fred,2007[26]	Mild/Severe	58.5	54.7	3	90	82	Met	Van	Method 1	(1)(2)(3)	High	Low
Wafa,2008[27]	Mild	71.0	NA	12	34	18	Met	Van	Method 1	(2)(3)	High	Unclear
Frank,2012[20]	Mild/Severe	60.5	49.0	12	128	16	Met	Van	Method 1	(1)(2)(3)	Moderate	Unclear
Jacques-a,2006[28]	Severe	NA	44.1	8	115	171	Met	Van	Method 2	(2)(3)	Moderate	Unclear
Enrico,2010[29]	Severe	53.0	50.0	4	19	7	Met	Van	Method 1	(1)(2)(3)	Moderate	Unclear
Wenisch,1996[30]	NA	41.0	53.2	4	31	31	Met	Van	Method 1	(3)	Moderate	Unclear
Ethan,2011[31]	Mild	12.1	48.7	8	37	37	Met	Van	Method 1	(1)	Moderate	Unclear
Sahil,2013[32]	Mild	2.3	54.3	12	69	6	Met	Van	Method 1	(1)(2)	Moderate	Unclear
**Mono vs. Combi**												
Danny,2006[10]	NA	69.0	41.0	4	20	19	Met	Met+Rif	Method 1	(1)(2)(3)	High	Low
Bass,2013[33]	Severe	65.8	NA	4	35	43	Van	Met+Van	Method 1	(1)(2)(3)	High	Unclear
Jacques-b,2006[28]	Severe	NA	44.1	8	115	36	Met	Met+Van	Method 2	(2)(3)	Moderate	Unclear
Jacques-c,2006[28]	Severe	NA	44.1	8	171	36	Van	Met+Van	Method 2	(2)(3)	Moderate	Unclear
Mihaela-a,2013[34]	NA	67.1	41.7	8	132	98	Met	Met+Van	Method 1	(2)	Moderate	Unclear
Mihaela-b,2013[34]	NA	67.1	41.7	8	76	98	Van	Met+Van	Method 1	(2)	Moderate	Unclear
Sapna-a,2014[35]	NA	60.0	57.5	6	54	13	Met	Met+Van	Method 1	(1)(2)	Moderate	Unclear
Sapna-b,2014[35]	NA	60.0	57.5	6	6	13	Van	Met+Van	Method 1	(1)(2)	Moderate	Unclear

Abbreviations: T: Treatment (Met or Mono); C: Control (Van or Combi); Met: Metronidazole; Van: Vancomycin; Rif: Rifampin; Mono: Monotherapy group; Combi: Combination therapy group; NA: Not available. Method 1: *C*. *difficile* toxin assay and/or a clinical diagnosis; Method 2: *C*. *difficile* toxin assay; (1): Rate of clinical cure; (2): Rate of CDI recurrence; (3): AEs.

### Efficacy outcomes

#### Rate of clinical cure

**Fig 2:**
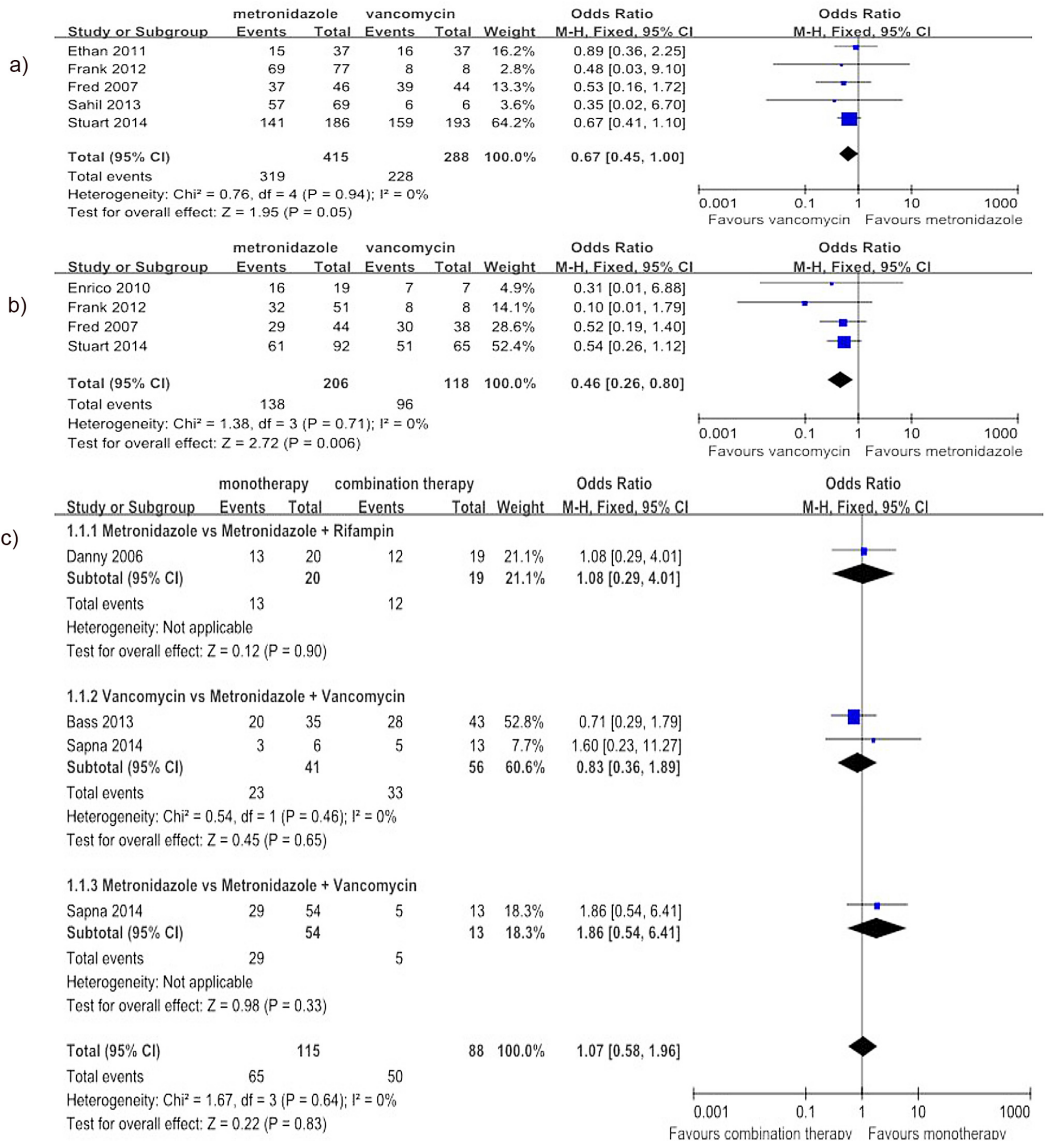
Forest plot of the rate of clinical cure (a: metronidazole vs. vancomycin for mild CDI; b: metronidazole vs. vancomycin for severe CDI; c: monotherapy vs. combination therapy). The vertical line indicates no difference between the groups. ORs are represented by diamond shapes, and 95% CIs are depicted by horizontal lines. Squares indicate point estimates, and the size of each square indicates the weight of the given study in the meta-analysis. M-H, Mantel-Haenszel fixed-effects model.

Regarding the comparison of metronidazole with vancomycin, the meta-analysis results suggested that there was no significant difference in the rate of clinical cure for the treatment of mild CDI (5 studies[13,20,26,31,32], 703 patients, OR = 0.67, 95% CI (0.45, 1.00), p = 0.05, I^2^ = 0%) (Fig 2A). However, the rate of clinical cure was lower for metronidazole than for vancomycin for the treatment of severe CDI (4 studies[13,20,26,29], 324 patients, OR = 0.46, 95% CI (0.26, 0.80), p = 0.006, I^2^ = 0%) (Fig 2B). Regarding the comparison of monotherapy with combination therapy, three articles were included[10,33,35], and the cases were divided into three subgroups according to therapeutic intervention used. The meta-analysis results did not show a significant difference in the rate of clinical cure between monotherapy and combination therapy (4 studies (one article included two separate studies[35]), 190 patients, OR = 1.07, 95% CI (0.58, 1.96), p = 0.83, I^2^ = 0%) (Fig 2C).

#### Rate of CDI recurrence

**Fig 3:**
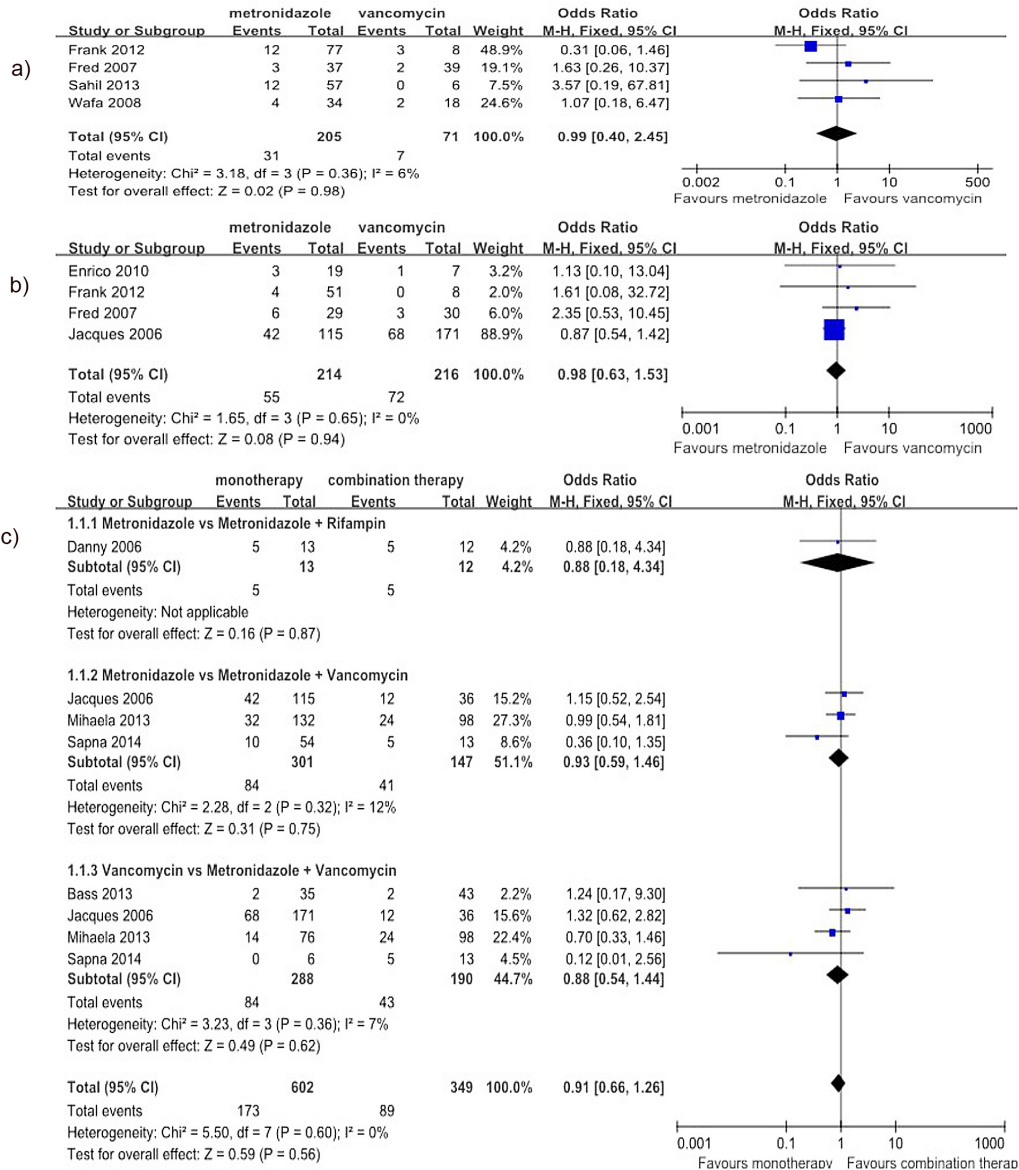
Forest plot of the rate of CDI recurrence (a: metronidazole vs. vancomycin for mild CDI; b: metronidazole vs. vancomycin for severe CDI; c: monotherapy vs. combination therapy). The vertical line indicates no difference between the groups. ORs are represented by diamond shapes, and 95% CIs are depicted by horizontal lines. Squares indicate point estimates, and the size of each square indicates the weight ofthe given study in the meta-analysis. M-H, Mantel-Haenszel fixed-effects model.

Regarding the comparison of metronidazole with vancomycin, the meta-analysis results did not show any significant difference in the rate of CDI recurrence for the treatment of mild CDI (4 studies[20,26,27,32], 276 patients, OR = 0.99, 95% CI (0.40, 2.45), p = 0.98, I^2^ = 6%) (Fig 3A) or severe CDI (4 studies[20,26,28,29], 430 patients, OR = 0.98, 95% CI (0.63, 1.53), p = 0.94, I^2^ = 0%) (Fig 3B). Regarding the comparison of monotherapy with combination therapy, five articles were included[10,28,33–35], and the cases were divided into three subgroups according to the therapeutic intervention. The meta-analysis results did not show any significant difference in the rate of CDI recurrence between monotherapy and combination therapy (8 studies (three articles each included two separate studies[28,34,35]), 804 patients, OR = 0.91, 95% CI (0.66, 1.26), p = 0.56, I^2^ = 0%) (Fig 3C).

### Safety outcomes

#### AEs

**Fig 4:**
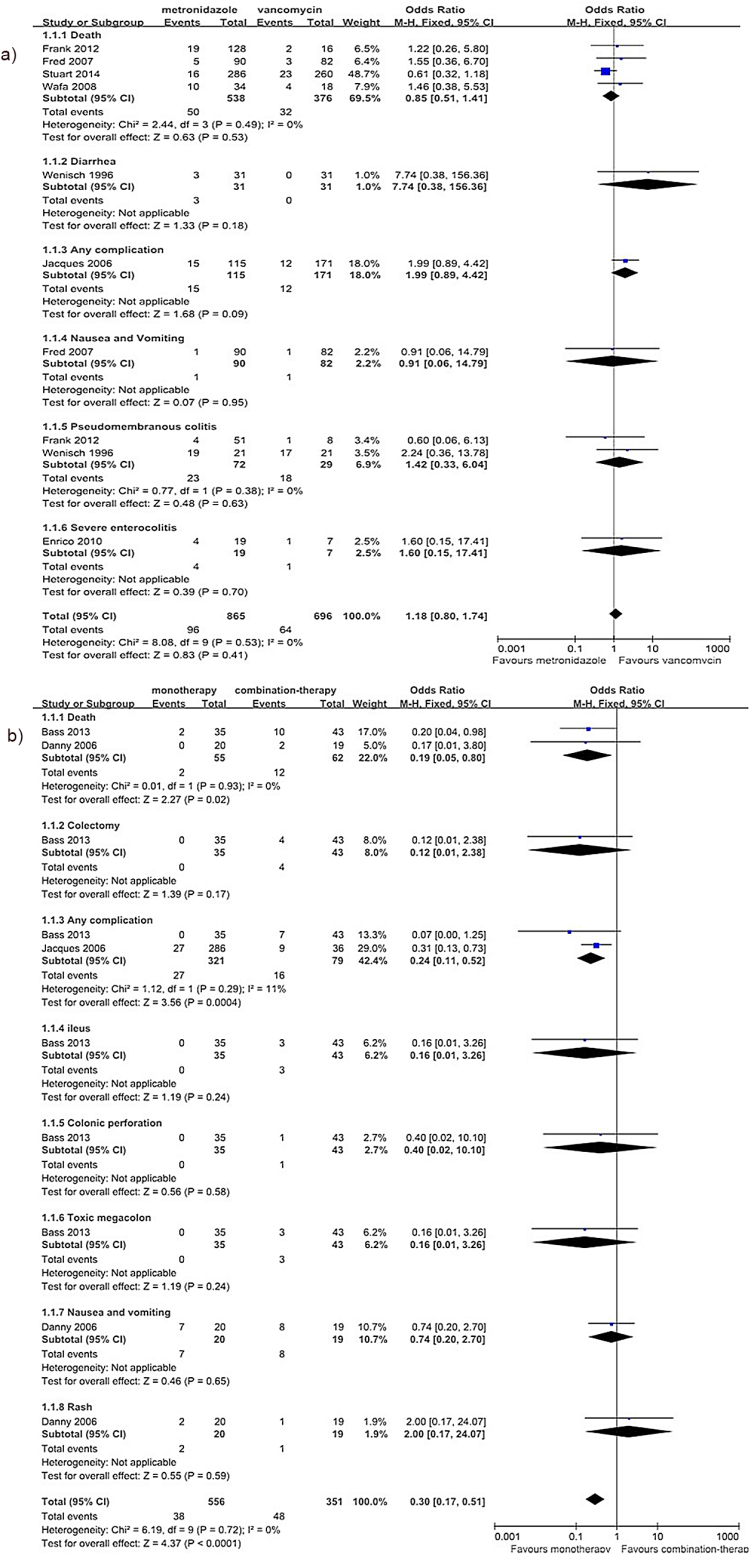
Forest plot of the rate of AEs (a: metronidazole vs. vancomycin; b: monotherapy vs. combination therapy). The vertical line indicates no difference between the groups. ORs are represented by diamond shapes, and 95% CIs are depicted by horizontal lines. Squares indicate point estimates, and the size of each square indicates the weight of the given study in the meta-analysis. M-H, Mantel-Haenszel fixed-effects model.

The reported AEs from the included studies consisted of death, colectomy, diarrhea, any complication, ileus, colonic perforation, nausea and vomiting, pseudomembranous colitis, toxic megacolon, rash and severe enterocolitis. We performed subgroup analysis according to the AEs. The meta-analysis results did not show any significant difference in the rate of AEs between metronidazole and vancomycin (the results from 7 studies were separated into six subgroups[13,20,26–30], 1330 patients, OR = 1.18, 95% CI (0.80, 1.74), p *=* 0.41, I^2^ = 0%) (Fig 4A). However, the rate of AEs was significantly lower for monotherapy than for combination therapy (the results from 3 studies were separated into eight subgroups [10,28,33], 439 patients, OR = 0.30, 95% CI (0.17, 0.51), p<0.0001, I^2^ = 0%) (Fig 4B); in fact, this rate was more than 4-fold higher for combination therapy than for monotherapy (46.9% vs.11.1%).

### Publication bias and sensitivity analyses

The shape of Begg’s funnel plot and the Egger’s test results (all p>0.05) did not demonstrate any evidence of publication bias (S1 File). Considering that meta-analysis showed little heterogeneity (I^2^ = 6%) between the studies for the comparison of the rate of CDI recurrence between metronidazole and vancomycin. Thus, we conducted sensitivity analyses of the subgroups from these studies according to explicit or inexplicit classifications of severity and follow-up duration to explore underlying sources of heterogeneity. These analyses showed that the primary results were not influenced by the inexplicit classification of severity or by the follow-up durations (S2 File).

## Discussion

The aim of this study was to compare the efficacy and safety of metronidazole monotherapy with vancomycin monotherapy and combination therapy in CDI patients. Based on the analyses according to disease severity and the treatment methods used, this study has revealed that metronidazole and vancomycin are equally effective for the treatment of mild CDI but that vancomycin is superior for severe CDI. Additionally, these findings suggest that combination therapy is not superior to monotherapy.

Regarding efficacy, the results of this meta-analysis demonstrated that the treatments were equivalent for mild disease but not for severe disease. Moreover, regarding the cost of the medications, metronidazole is less expensive than vancomycin[41], and oral vancomycin may be more likely to promote the generation and overgrowth of VRE than metronidazole[5]. As a result, metronidazole is recommended for treating mild CDI, and vancomycin is recommended for treating severe CDI. This meta-analysis also demonstrated that combination therapy might not be more effective than monotherapy. Regarding safety, although there was no statistically significant difference in the rate of AEs between metronidazole and vancomycin, there was an increase in the rate of AEs in association with combination therapy, and the combination therapy group experienced more fatal AEs, such as death and complications. Therefore, monotherapy appears to be safe and well tolerated. Nonetheless, it is worth mentioning that because of the limited number of included studies, we did not compare monotherapy with combination therapy for all four categories of disease severity. Moreover, our results may have been influenced by the medical conditions or treatment (e.g., nasogastric feeds[10]) of the patients receiving combination therapy, who may have suffered from more serious illnesses than those receiving monotherapy; therefore, these influences may have been associated with the increased risk of experiencing AEs or developing complications among those receiving combination therapy.

Our study has several limitations. First, although the quality of the majority of the studies was high or medium and although the total sample size was sufficient, the sample size of each subgroup was relatively small and was thus susceptible to false-positive or false-negative results. Second, although sensitivity analyses indicated that the primary results were not influenced by the inexplicit classification of severity or by the follow-up duration, we roughly determined disease severity based on the proportion of patients, which potentially resulted in bias. For example, if 77% of the patients had mild CDI[27], we categorized all of the patients into the mild CDI group. Moreover, the follow-up duration for CDI treatment varied greatly (from 3–12 weeks) among the different studies, which may have resulted in bias. Third, because very few articles presented research on combination therapy, we could not compare the subgroups according to disease severity between monotherapy and combination therapy; therefore, the general patient conditions may have caused prescription bias. Fourth, only studies published in English were included, which might have rendered the results vulnerable to bias related to language and ethnicity. Fifth, additional data on combination therapy using other novel antibiotics are needed.

In conclusion, these findings have important clinical implications and support the recommendations of the current CDI treatment guidelines[3,4]. However, validation of the routine use of combination therapy may require additional evidence. To compensate for the shortcomings of our study, further large-scale clinical trials and well-designed research studies are needed to identify more effective therapies for patients with CDI.

## Supporting Information

S1 PRISMA Checklist PRISMA checklist for the meta-analysis. (DOC)

S1 Fig Risk of bias summary. Review about each risk of bias item for each included study.
(TIF)

S1 File Publication bias. (DOC)

S2 File Sensitivity analysis. (DOC)

S1 Table Search strategy. (DOC)

S2 Table The quality assessment of evidence for each included study by the modified JADAD score. (DOC)
