# Supplementary material for: Efficacy and Safety of Metronidazole Monotherapy versus Vancomycin Monotherapy or Combination Therapy in Patients with Clostridium difficile Infection: A Systematic Review and Meta-Analysis
Source: PLoS One. 2015 Oct 7;10(10):e0137252. doi: 10.1371/journal.pone.0137252 (PMC4621873; doi:10.1371/journal.pone.0137252)
Supplement: S1 File — (DOC) [file pone.0137252.s003.doc]

**S1 File.** Publication bias.

A B

C D

**Fig 1.** Begg's funnel plot. Each dot represents a separate study.

A: Rate of clinical cure (metronidazole vs. vancomycin: mild+severe CDI); B: rate of CDI recurrence (metronidazole vs. vancomycin: mild+severe CDI); C: rate of clinical cure (monotherapy vs. combination therapy); and D: rate of CDI recurrence (monotherapy vs. combination therapy).

**Note:** Visual inspection of the funnel plot shown in Fig 1-A revealed slight asymmetry; however, evaluation of Fig 1-A by Egger's test did not reveal any evidence of publication bias. A possible reason for this finding was heterogeneity among studies rather than publication bias. In addition, the dots in Fig 1-A reveal an apparent trend between small study size and increased effect of vancomycin, but the large studies show much more moderate effects. This finding may be attributed to better responses of the more severe patients in the smaller studies to vancomycin.

**Table 1.** Egger's test of Fig 1-A.

Std_Eff | Coef. Std. Err. t P>|t| [95% Conf. Interval]

slope | -.2118418 0.1521095 -1.39 0.206 -.5715236 0.14784

bias | -.6804182 0.3016252 -2.26 0.059 -1.393648 0.032812

p=0.059>0.05

**Table 2.** Egger's test of Fig 1-B.

Std_Eff | Coef. Std. Err. t P>|t| [95% Conf. Interval]

slope | -.2814308 0.2818686 -1.00 0.357 -.9711385 0.4082769

bias | 0.4975951 0.4806967 1.04 0.341 -.6786274 1.673818

p=0.341>0.05

**Table 3.** Egger's test of Fig 1-C.

Std_Eff | Coef. Std. Err. t P>|t| [95% Conf. Interval]

slope | -1.019804 0.8153404 -1.25 0.338 -4.527931 2.488323

bias | 1.798743 1.309577 1.37 0.303 -3.835913 7.433399

p=0.303>0.05

**Table 4.** Egger's test of Fig 1-D.

Std_Eff | Coef. Std. Err. t P>|t| [95% Conf. Interval]

slope | 0.3541301 0.3299837 1.07 0.324 -.453311 1.161571

bias | -1.020467 .6932502 -1.47 0.191 -2.716789 0.675855

p=0.191>0.05
