# Supplementary material for: Efficacy and Safety of Metronidazole Monotherapy versus Vancomycin Monotherapy or Combination Therapy in Patients with Clostridium difficile Infection: A Systematic Review and Meta-Analysis
Source: PLoS One. 2015 Oct 7;10(10):e0137252. doi: 10.1371/journal.pone.0137252 (PMC4621873; doi:10.1371/journal.pone.0137252)
Supplement: S2 File — (DOC) [file pone.0137252.s004.doc]

**S2 File.** Sensitivity analyses.


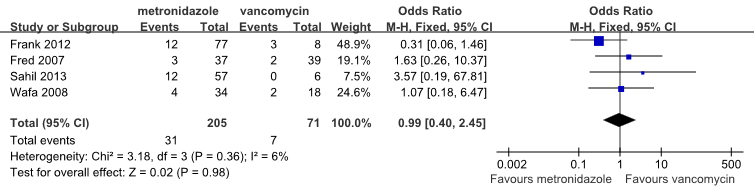


A


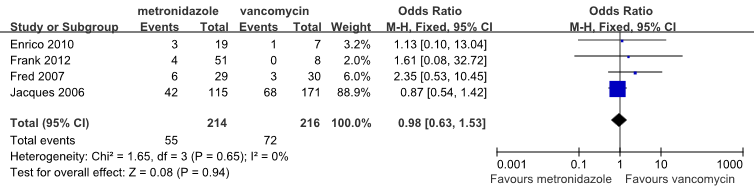


B

**Fig 1**. Rate of CDI recurrence for metronidazole vs. vancomycin (non-subgroup analysis).

A: mild CDI; B: severe CDI.


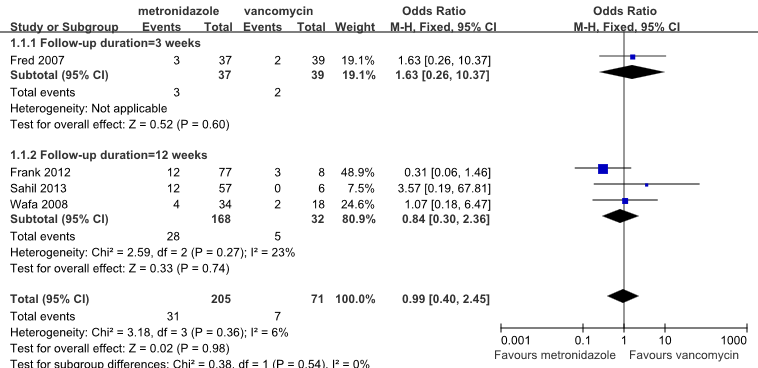


A


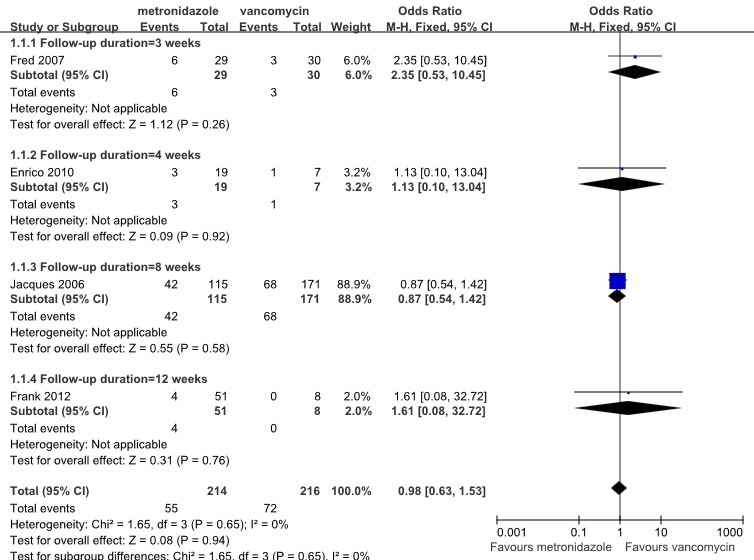


B

**Fig 1-1.** Rate of CDI recurrence for metronidazole vs. vancomycin (subgroup analysis

according to follow-up duration). A: mild CDI; B: severe CDI.


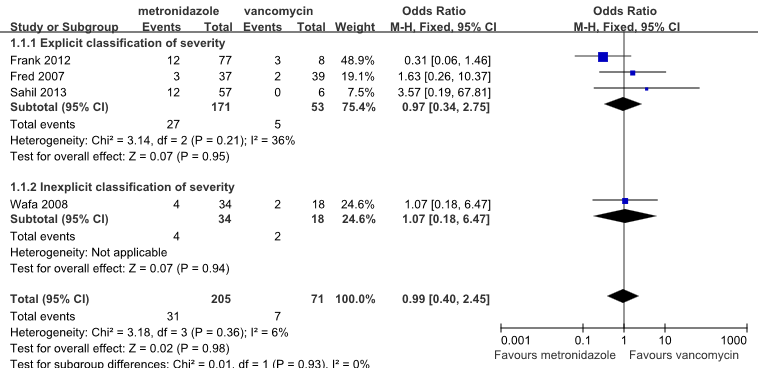


A

**Note:** No studies with inexplicit classification of severity were included in the B group (severe CDI).

**Fig 1-2.** Rate of CDI recurrence for metronidazole vs. vancomycin (subgroup analysis according to explicit or inexplicit classification of severity). A: mild CDI.
