# Supplementary material for: Efficacy and Safety of Metronidazole Monotherapy versus Vancomycin Monotherapy or Combination Therapy in Patients with Clostridium difficile Infection: A Systematic Review and Meta-Analysis
Source: PLoS One. 2015 Oct 7;10(10):e0137252. doi: 10.1371/journal.pone.0137252 (PMC4621873; doi:10.1371/journal.pone.0137252)
Supplement: S1 Table — (DOC) [file pone.0137252.s005.doc]

S1 Table. Search strategy.

| **PubMed:** 552 records (up to November 2014)  ((((Pseudomembranous enterocolitis[MeSH Terms]) OR Antibiotic-associated diarrhea[MeSH Terms]) OR Antibiotic-associated colitis[MeSH Terms]) OR Clostridium difﬁcile[MeSH Terms]) AND ((vancomycin[MeSH Terms]) OR metronidazole[MeSH Terms]) |
| --- |
| **Embase:** 420 records (up to November 2014)  'pseudomembranous enterocolitis' OR 'antibiotic-associated diarrhea' OR 'antibiotic-associated colitis' OR 'clostridium difﬁcile' AND ('metronidazole' OR 'vancomycin') |
| **Web of Science:** 353 records (up to November 2014)  TS=("pseudomembranous enterocolitis" OR "antibiotic-associated diarrhea" OR "antibiotic-associated colitis" OR "clostridium diffile" ) AND TS=(metronidazole OR vancomycin) |
| **Cochrane Library:** 108 records (up to November 2014)  "clostridium diffile":ti,ab,kw or "antibiotic-associated colitis":ti,ab,kw or "pseudomembranous enterocolitis":ti,ab,kw or "antibiotic-associated diarrhea":ti,ab,kw and metronidazole or vancomycin:ti,ab,kw |
| **CNKI (China National Knowledge Infrastructure):** 207 records (up to November 2014)  ( ( ( ( SU=clostridium diffile OR SU=pseudomembranous enterocolitis ) OR SU=antibiotic-associated colitis ) OR SU=antibiotic-associated diarrhea ) AND ( SU=metronidazole OR SU=vancomycin ) ) |
| **WANFANG DATA (another** **Chinese database):** 175 records (up to November 2014)  [Topic search:("clostridium diffile") + Topic search:("pseudomembranous enterocolitis") + Topic search:("antibiotic- associated colitis") + Topic search:("antibiotic-associated diarrhea") * Topic search:("metronidazole" + "vancomycin") * Date:-2014](http://librarian.wanfangdata.com.cn/javascript:copyToClipboard() |
| **VIP (Chinese Scientific Journals Database):** 55 records (up to November 2014)  1.Title/keywords=clostridium diffile OR Title/keywords=pseudomembranous enterocolitis OR Title/keywords=antibiotic- associated colitis OR Title/keywords=antibiotic-associated diarrhea AND Title/keywords=metronidazole (21 records)  2.Title/keywords=clostridium diffile OR Title/keywords=pseudomembranous enterocolitis OR Title/keywords=antibiotic- associated colitis OR Title/keywords=antibiotic-associated diarrhea AND Title/keywords=vancomycin (34 records)  3.1 and 2 |
