# Supplementary material for: Efficacy and Safety of Metronidazole Monotherapy versus Vancomycin Monotherapy or Combination Therapy in Patients with Clostridium difficile Infection: A Systematic Review and Meta-Analysis
Source: PLoS One. 2015 Oct 7;10(10):e0137252. doi: 10.1371/journal.pone.0137252 (PMC4621873; doi:10.1371/journal.pone.0137252)
Supplement: S2 Table — (DOC) [file pone.0137252.s006.doc]

**S2 Table.** Quality appraisal of studies included in the meta-analysis.

| Study | Random  allocation | Concealment  schemes | Blinding | Drop-  out | Integrity of  the results | Selective  report | Jadad scale |
| --- | --- | --- | --- | --- | --- | --- | --- |
| Danny 2006[10]  Stuart 2014[13]  Fred 2007[26]  Wafa 2008[27]  Bass 2013[33]  Frank 2012[20]  Jacques 2006[28]  Enrico 2010[29]  Wenisch 1996[30]  Ethan 2011[31]  Sahil 2013[32]  Mihaela 2013[34]  Sapna 2014[35] | Yes  Yes  Yes  Yes  Yes  UA  UA  Yes  Yes  UA  UA  UA  UA | UA  UA  UA  UA  UA  UA  UA  UA  UA  UA  UA  UA  UA | S-B  D-B  D-B  UA  UA  UA  UA  UA  UA  UA  UA  UA  UA | Yes  Yes  Yes  Yes  Yes  UA  UA  UA  UA  UA  Yes  UA  UA | Yes  Yes  Yes  Yes  Yes  Yes  Yes  Yes  Yes  Yes  Yes  Yes  Yes | UA  UA  UA  UA  UA  UA  UA  UA  UA  UA  UA  UA  UA | 6  6  6  5  5  3  3  3  3  3  3  3  3 |

Abbreviations: UA:Unclear; S-B:single-blinded; D-B: double-blind.

**Jadad scale:** Points were determined as follows, I. generation of allocation sequence (computer-generated random numbers, 2 points; not described, 1 point; inappropriate method,0 point); II. allocation concealment (central randomization, sealed envelopes or similar, 2 points; not described, 1 point; inappropriate or unused, 0 point); III. blindness (identical placebo tablets or similar, 2 point; inadequate or not described, 1 point; inappropriate or no double blinding, 0 point); IV. withdrawals and drop-outs (numbers and reasons are described, 1 point; not described, 0 point). The Jadad scale score ranges from 1 to 7; higher score indicates better RCT quality. If a study had a modified Jadad score >4 points, it was considered to be of high quality; if the score was 3-4 points, it was of moderate quality; and if the score was <3 points, it was of low quality.
